# Manipulation and treatment of textiles and their relationship with healthcare-associated infections: a scoping review

**DOI:** 10.1590/1980-220X-REEUSP-2025-0430en

**Published:** 2026-04-27

**Authors:** Talita Tavares Della Motta, Adriane Reis Barletta Canicoba, Alessandra Yuri Takehana de Andrade, Ramon Antônio Oliveira, Renata Cristina de Campos Pereira Silveira, Vanessa de Brito Poveda

**Affiliations:** 1 Universidade de São Paulo, Escola de Enfermagem, Departamento de Enfermagem Médico-cirúrgica, São Paulo, SP, Brazil.; 2 Universidade de São Paulo, Escola de Enfermagem de Ribeirão Preto, Departamento de Enfermagem Fundamental, Ribeirão Preto, SP, Brazil.

**Keywords:** Textiles, Cross Infection, Bedding and Linens, Clothing, Infection Control, Têxteis, Infecção Hospitalar, Roupas de Cama, Mesa e Banho, Vestuário, Controle de Infecções

## Abstract

**Background:**

Textiles are essential and indispensable materials in healthcare, comprising a wide range of items such as patient clothing, staff uniforms, bed linen, curtains, and reusable sterile drapes. However, these materials can become vehicles for contamination, contribute to the spread of microorganisms, or even increase the rates of healthcare-associated infections (HCAIs).

**Objective:**

To map the available evidence in the scientific literature regarding the handling, processing, and contamination of textiles and the occurrence of HCAIs.

**Method:**

A scoping review was conducted following the JBI recommendations. The following databases were consulted: Web of Science, PubMed/Medline, CINAHL, LILACS, EMBASE, SCOPUS, and SCIELO. Grey literature was searched via the CAPES Theses and Dissertations Catalogue, MEDNAR, and Google Scholar. The duplicate removal, independent screening, data extraction, and synthesis were performed by two reviewers, and any discrepancies were resolved by a third evaluator. Observational or experimental studies, published in any language and without time restrictions were included. Data were presented through descriptive synthesis and tables containing relevant information.

**Results:**

Twenty studies were included, with case series being the most common study design (14; 70%). The main theme involved outbreak investigations, with *Bacillus cereus* (7; 35,0%) emerging as the most significant microorganism contaminating textiles that come into contact with patients’ skin. Contamination was linked to handling techniques, cleaning, processing, and storage practices. The use of copper oxide was suggested as a strategy in fabric production aimed at reducing contamination and HCAI rates.

**Conclusion:**

Controlling textile contamination requires an institutional commitment to active surveillance, quality hospital processes, and adherence to strict protocols, ensuring safe handling, processing, and storage, thereby reducing negative impacts on patient health and the effectiveness of the services provided.

## RESUMO

**Contexto:**

Os têxteis são materiais essenciais e indispensáveis ao cuidado em saúde, constituindo uma diversidade de itens como o vestuário dos pacientes, uniformes dos profissionais, roupas de cama, cortinas e campos estéreis reprocessáveis. Entretanto, esses materiais podem tornarse veículos de contaminação, colaborar na disseminação de microrganismos, ou mesmo elevar as taxas de infecções relacionadas à assistência à saúde (IRAS).

**Objetivo:**

Mapear as evidências disponíveis na literatura científica a respeito das formas de manipulação, processamento e contaminação de têxteis e a ocorrência das IRAS.

**Método:**

Revisão de escopo segundo as recomendações do JBI. Foram consultadas as bases de dados Web of Science, PubMed/Medline, CINAHL, LILACS, EMBASE, SCOPUS e SCIELO. A busca na literatura cinzenta incluiu as bases Catálogo de Teses e Dissertações – CAPES, MEDNAR e Google Acadêmico. A estratégia de busca foi estruturada utilizando termos e descritores a partir dos Medical Subject Headings (MESH) e dos Descritores em Ciências da Saúde (DECS) e os registros foram gerenciados no Rayyan, com remoção de duplicatas e triagem independente por dois revisores. As divergências foram solucionadas por um terceiro avaliador. A extração e a síntese dos dados foram realizadas por dois revisores, resultando em uma síntese descritiva e em quadros narrativos com as informações relevantes. Foram incluídos estudos primários, observacionais ou experimentais, com delineamento quantitativo, publicados em qualquer idioma e sem delimitação temporal.

**Resultados:**

Foram incluídos 20 estudos, com destaque para o delineamento de série de casos (14; 70%). O tema principal envolveu a investigação de surtos, sendo o *Bacillus cereus* (7; 35,0%) o mais relevante na contaminação de têxteis que entram em contato com a pele dos pacientes. Os casos de contaminação estiveram relacionados às técnicas de manipulação, higienização, processamento e armazenamento. O uso de óxido de cobre foi sugerido como estratégia para a fabricação de tecidos com o objetivo de reduzir a contaminação e as taxas de IRAS.

**Conclusão:**

O controle da contaminação de têxteis requer um compromisso institucional com a vigilância ativa, a qualidade dos processos hospitalares e a adesão a protocolos rigorosos, assegurando manipulação, processamento e armazenamento seguros, reduzindo os impactos negativos sobre a saúde dos pacientes e a eficácia dos serviços prestados.

## INTRODUCTION

Healthcare-associated infections (HCAIs) are among the most prevalent adverse events in hospital settings worldwide, and they have significant repercussions on the patient’s clinical, emotional, and functional condition, in addition to generating economic impacts on healthcare institutions and health systems^(1)^. According to the World Health Organization (WHO), approximately 22 out of every 100 patients admitted to acute care hospitals acquire at least one HCAI, with patients from low- and middle-income countries presenting nearly double the number of cases compared to high-income countries (15 vs. 7 affected patients per 100 admissions, respectively)^(2)^.

The etiology of HCAIs is multifactorial, involving intrinsic patient factors and aspects related to care processes, such as environmental conditions and the characteristics of materials and devices used^(3,4)^.

Textiles such as bed linen, uniforms, curtains, and reusable sterile drapes are essential components of healthcare environments and are in direct contact with both patients and healthcare professionals. While these materials are used for the care and protection of patients and staff, they may at times serve as vectors of contamination and contribute to the dissemination of microorganisms, ultimately increasing the rates of HCAIs. This is primarily due to the porous nature of textile fibers, which facilitates microbial growth, promotes biofilm formation, and allows for the contamination of other surfaces through contact^(5,6,7)^.

Even textiles that do not come into direct contact with the bodies of patients or healthcare professionals, such as privacy curtains between hospital beds, require special and frequent care to ensure adequate routine decontamination in the institutions where they are used. These items often harbor a substantial microbial load, including multidrug-resistant microorganisms that play a significant role in the pathogenesis of HCAIs^(8)^.

In this context, hospital textiles can act as potential reservoirs of microorganisms associated with HCAIs, especially in critical areas such as Intensive Care Units, emergency departments, and surgical wards, harboring clinically relevant pathogens such as *Staphylococcus aureus* (including Methicillin Resistant *Staphylococcus aureus-MRSA*), *Escherichia coli*, *Pseudomonas aeruginosa*, *Clostridioides difficile*, and *Acinetobacter baumannii*, which are capable of surviving for prolonged periods on fabrics, with their behavior varying according to fiber type, humidity, and environmental conditions^(9)^.

Thus, the characteristics of the textile, the adhesion of pathogenic microorganisms to the weave, the cleaning technique required for each textile according to its composition, and the type of contact with the skin of healthcare service users must all be taken into account. In this context, a study analyzing bacterial adhesion and persistence in hospital textiles demonstrated that microorganisms such as bacteria and fungi can survive for approximately 26 days on various types of material, including cotton, polypropylene, polyester, viscose, silk, and wool fibers. Furthermore, the duration of microbial persistence and the bacterial load present in the textile fibers were associated with the physicochemical characteristics, nanoroughness of the fabric, and surface properties that influence the adhesion capacity of each microorganism to the fiber^(10)^.

An *in vitro* study using porcine skin, which has characteristics similar to human skin, demonstrated that it is possible to transfer multidrug-resistant microorganisms from the dry surface of a textile to the animal’s skin^(11)^. The results of another laboratory-based investigation indicated an increase in tissue contamination rates when microorganisms had been recently inoculated into the textile, with transfer being five times higher when the fabric was wet or when it was rubbed against the skin^(12)^. In light of these findings, technologies have been developed for the production and treatment of textiles, such as fabrics impregnated with antimicrobial agents which have shown effectiveness in controlling contamination, reducing microbial load, and decreasing the occurrence of HCAIs. Nevertheless, important gaps remain, including limited data on the behavior of microbial communities in textiles used in real healthcare settings, as well as the potential for antimicrobial resistance and cytotoxicity^(5,9,13,14,15)^.

Thus, in February 2025, searches for potential previous reviews were conducted in the EPISTEMONIKOS, Cochrane Library, Open Science Framework, and PubMed/Medline electronic databases and no records were found of reviews using only studies that associated the presence of contaminated textiles with HCAI rates^(7,10)^.

Therefore, considering the absence of relevant reviews on the topic, despite the potential importance of textiles as probable contamination vehicles in healthcare services, it is mandatory to identify the existing evidence, its quality, and the potential gaps related to textiles as fomites in the etiology of HCAIs, the factors that contribute to the contamination of these materials, and the appropriate handling and hygiene strategies. In this regard, the results of the present study may support managerial decision-making, improve the practices of cleaning and nursing teams, and strengthen the strategies of infection control services.

### Objective

To map the evidence available in the scientific literature regarding the relationship between the handling and processing of textile materials, the presence of contamination by pathogenic microorganisms, and the occurrence of HCAIs in hospital healthcare settings.

## METHOD

This is a scoping review, following the JBI Manual for Evidence Synthesis recommendations and reported according to the Preferred Reporting Items for Systematic Reviews and Meta-analyses Scoping Review (PRISMA-Scr)^(16)^. The research question was formulated based on the PCC acronym (P: population of interest; C: concept; C: context), which, respectively, refers to textiles used in healthcare (“Population”), contamination and occurrence of HCAIs (“Concept”), and hospital healthcare settings (“Context”) described in Chart 1. Thus, the question addressed by this review is: What is the evidence regarding the relationship between handling and processing of textiles and their contamination, and the occurrence of HCAIs in hospital healthcare settings? The protocol was registered on the Open Science Framework (OSF) platform, DOI https://doi.org/10.17605/OSF.IO/GNC8M.

**Chart 1:** Inclusion and exclusion criteria by PCC – Caraguatatuba, SP, Brazil, 2025.

	PCC acronym	Inclusion criteria	Exclusion criteria
P	“Population”	Textiles used in healthcare	Non-woven fabric (NWF) or disposable materials
C	“Concept”	Studies addressing textile contamination, methods of handling and treatment, and the occurrence of HCAIs in hospital healthcare settings were included.	Those addressing textiles contamination without a specific association with HCAIs
C	“Context”	Hospital healthcare settings	Studies conducted in non-hospital contexts (such as dentistry or veterinary medicine)

Primary, quantitative studies, with no limitations regarding language or publication period, including case reports, case series, cross-sectional studies, cohort studies, case-control studies, before-and-after studies, experimental or quasi-experimental studies, and clinical guidelines, were included. Additionally, studies addressing textile contamination, methods of handling and treatment, and the occurrence of HCAIs in hospital healthcare settings were included. Studies that exclusively focused on the use of non-woven fabric (NWF) or disposable materials, those addressing textiles contamination without a specific association with HCAIs, studies conducted in non-hospital contexts (such as dentistry or veterinary medicine), animal studies, *in vitro* studies, letters to the editor, theoretical essays, and abstracts presented at conferences were excluded.

### Evidence Collection

The terms or descriptors used for the research in the indexed databases were selected from the Medical Subject Headings (MeSH) and Health Sciences Descriptors (DeCS), using both controlled and uncontrolled descriptors combined to ensure a comprehensive search, with the assistance of a specialized librarian^(17)^ (Supplement 1)^(18)^.

The search for studies was conducted in April and May 2024 in the following databases: Web of Science, PubMed/Medline, Cumulative Index to Nursing and Allied Health Literature (CINAHL), Latin American and Caribbean Literature in Health Sciences (LiLACS), EMBASE, SCOPUS, and Scientific Electronic Library Online (SciELO), with no time or language restrictions. The search in the grey literature was conducted in July 2024 in the CAPES and MEDNAR Theses and Dissertations Catalogues, as well as in Google Scholar, reviewing all records. Finally, the references of the selected studies were also reviewed to identify relevant studies.

### Screening of Retrieved Records

The results retrieved from the databases were imported into the Rayyan Intelligent Systematic Reviews platform. Following the removal of duplicates, titles and abstracts were screened in accordance with the predefined inclusion and exclusion criteria, without the use of artificial intelligence tools. Full-text articles of the selected studies were subsequently obtained and independently assessed by two reviewers, employing the platform’s blinding feature. Any disagreements during the selection process were resolved by a third reviewer.

### Data Synthesis

Data were extracted independently by two reviewers. In cases of uncertainty or disagreement, a third expert was consulted to reach consensus. The synthesis of the data was conducted descriptively, and narrative tables were compiled to present the relevant information in a structured manner.

## RESULTS

**Figure 1:**
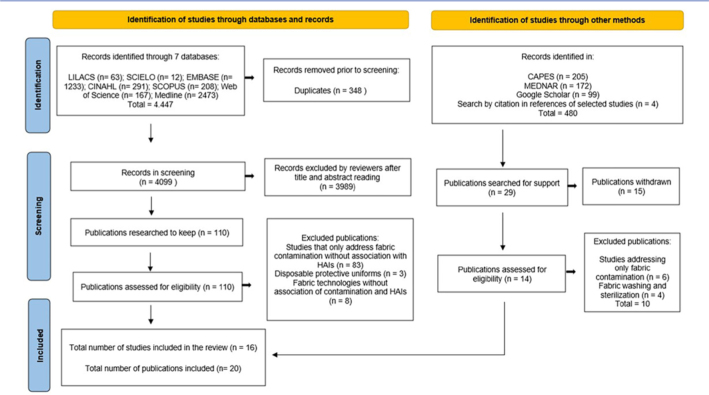
Study selection flowchart adapted from PRISMA-ScR – Caraguatatuba, SP, Brazil, 2025.

A total of 4,447 records were retrieved from the assessed databases. Following the removal of duplicates, 4,099 manuscripts remained for title and abstract screening. Of these, 108 articles were subjected to full-text evaluation, resulting in the selection of 16 studies for inclusion in the review. Concurrently, 482 potentially eligible records were identified through grey literature sources and four met the inclusion criteria. Thus, the final sample comprised 20 studies, which were included in this scoping review (Figure 1).

### General Characteristics of the Included Studies

The final sample comprised 20 studies published between 1981 and 2020, with the case series design employed in 12^(19,20,21,22,23,24,25,26,27,28,29,30)^. The majority of studies were published in English (n = 18; 90%)^(19,20,21,22,23,24,25,26,27,29,28,29,30,31,32,33,34,35,36,37)^, with one in Italian (5%)^(38)^ and one in Japanese (5%)^(28)^. The countries with the highest number of publications on the topic were the United States (n = 5; 25%)^(25,29,31,34,37)^, the United Kingdom (n = 5; 25%)^(19,20,21,24,26)^, Japan (n = 4; 20%)^(22,23,28,32)^, China (n = 2; 10%)^(27,35)^, and Israel (n = 2; 10%)^(33,36)^, followed by the Netherlands (n = 1; 5%)^(30)^ and Italy (n = 1; 5%)^(38)^(Supplement 2)^(18)^.

Regarding the main subject, the majority of studies (n = 14; 70.0%)^(19,20,21,22,23,24,25,26,27,28,29,30,32,35)^ investigated the relationship between the presence of textiles contaminated by microorganisms and the occurrence of HCAIs in healthcare units. Four studies (20.0%)^(33,34,36,37)^ addressed the use of biocidal compounds such as copper oxide in the manufacture of fabrics as a strategy to reduce contamination and HCAIs rates. Two additional studies (10.0%) explored other textile-related strategies, including the evaluation of the impact of private gown use on HCAIs rates in a neonatal intensive care unit^(31)^, and a comparison of reusable cloth gowns and drapes with disposable drapes in relation to surgical site infection rates^(38)^ (Supplement 2)^(18)^.

Some studies (n = 3; 15%)^(22,24,29)^ reported seasonal outbreaks of infection linked to climate and temperature variation, particularly during the warmer months of the year, which promote the proliferation and survival of microorganisms that thrive in heat and humidity. Additionally, the contamination observed in textiles was associated with a range of outcomes, most notably bacteraemia, skin infections, and respiratory infections, leading to either fatal or non-fatal adverse events (Chart 2).

**Chart 2:** Type of textile, identified microorganisms, resistance profile, origin of contamination and control strategy – Caraguatatuba, SP, Brazil, 2025.

Study	Type of microorganism (Microorganism)	Occurrence of multidrug resistance (antibiotics)	Type of contaminated fabric	Fabric in contact with the skin	Origin of contamination	Used/suggested strategy for treatment/processing of textiles
Birch *et al*. [18]	Bacterium *(B. cereus)*	NI	Clean diapers	Yes	Contamination of the washing machine due to the use of rice starch	Control of fabric washing equipment and post-wash surveillance cultures.
Barrie *et al*. [19]	Bacterium *(B. cereus)*	NI	Bed linen	Yes	Bed linen used and stored damp in plastic bags, at high ambient temperatures	Washing machine sanitation using hypochlorite for 18 hours and steam (temperature of 82°C). Routine microbiological monitoring.
Das *et al*. [22]	Bacterium *(A. baumanii)*	Yes (Carbapenem)	Privacy curtain	No	Probable contamination by hands	Increased frequency of cleaning the environment with hypochlorite (1000 ppm). Curtains are changed twice a week. Restricted use of Meropenem in the units. Hand hygiene with soap and water or aqueous chlorhexidine OR alcohol. Reminders to use gloves and aprons for direct contact with patients with transmission risk.
Shiomori, *et al*. [23]	Bacterium *(MRSA)*	Yes (Methicillin)	Bed linen	Yes	Dispersion of microorganisms during bed making with contaminated sheets	Continuing education on bed cleaning for all medical and nursing staff.
Dohmae *et al*. [24]	Bacterium *(B. cereus)*	Yes (Ciprofloxacin/erythromycin/tetracycline/gentamicin/minocycline)	Towels	Yes	Ineffective sanitation process	Use of specific substances in washing fabrics used by patients colonized by relevant pathogenic microorganisms, such as the use of sodium hypochlorite in eliminating Bacterium *B. cereus*.
Sasahara *et al.* [25]	Bacterium *(B. cereus)*	NI	Bed linen: sheets and towels	Yes	Washing machine contamination due to the use of recycled water and poor maintenance	Autoclaved hospital sheets. Washing machine sanitation with powerful alkaline detergent. Water recycling suspension for washing and rinsing. In addition, hospital staff were required to wear gloves while handling intravenous devices, not only during puncture, but also during the administration of intravenous therapy.
Hosein *et al*. [26]	Bacterium (*B. cereus*)	NI	Crib bed linen: blanket, sheet, pillowcase	Yes	Ineffective sanitation process	Increased water flow rate. Routine microbiological monitoring.
Duffy *et al.* [27]	Fungus (*Rhizopus delemar*)	NI	Bed linen: sheets, blankets, pillowcases, towels, and patient gowns	Yes	Contamination in the laundry or during delivery	Change in the bed linen supply company. Structural changes in delivery. Disposal of old hospital linen. Cleaning and disinfection of bed linen storage areas. Temporary use of autoclaved bed linen for patients considered to have a high risk of infection.
Mahida *et al.* [29]	Bacteria (*Streptococcus A)*	NI	Privacy curtains between beds	No	Cross-contamination through contact with professionals’ hands	Replace curtains with easy-to-clean materials. Keep track of how often you change and wash curtains.
Cheng *et al.* [30]	Fungi *(Rhizopus microsporus Zigomicetos)*	NI	Bed linen	Yes	Poor condition of the laundry – Dirt on wall fans, ceiling air duct outlets and the surface of ironing machines – Storage: damp and hot packaged sheets	Routine microbiological monitoring. Cleaning and disinfection of the unit and delivery vehicles, including surfaces and equipment. Maintenance of washing machines (calibration of temperature sensors, humidity control during drying and packaging processes). Structural changes: clear segregation between clean and dirty areas to avoid cross-contamination of processed items. Checking of bed linen storage conditions (temperature and humidity). Routine changes: consumption of bed linen on a “first in, first out” basis. Physicians should maintain a high index of suspicion for early diagnosis and treatment of zygomycosis in immunosuppressed patients.
Itoga *et al*. [31]	Bacterium *(B. cereus)*	NI	Towels	Yes	Ineffective sanitation process	Heat treatment processes above 160 °C, use of high-level disinfectants (peracetic acid and sodium hypochlorite) during washing. Microbiological monitoring during receipt of clothing/at the end of hygiene processes.
Teal *et al*. [33]	Fungi (*Rhizopus*)	NI	Bed linen	Yes	Contamination of the bed linen transport carts	Implementation of a routine for emptying and cleaning bed linen changing carts with disinfectant detergent registered by the Environmental Protection Agency (EPA). Surveillance cultures. Change in the bed linen supply company.
Cheng *et al.* [34]	Bacterium (*B. cereus)*	NI	Bed linen	Yes	Direct inhalation and skin contact with contaminated items	Change in the bed linen supply company. Complete drying of the fabric before storage.
Boonstra *et al*. [37]	Bacterium (*K. pneumoniae – ESBL*)	Yes (cefotaxime and/or ceftazidime, tobramycin, amikacin, kanamycin, ciprofloxacin and norfloxacin)	Bed linen	Yes	Washing machine contamination due to improper use	Isolation of contaminated patients. Adequate operation of the machine temperature during the washing process.

Most of the contaminated textiles were items in direct contact with patients’ skin, such as bed linen, sheets, pillowcases, blankets, nappies, and bath towels (n = 13; 65%)^(19,20,21,22,23,24,25,27,28,29,30,32,35)^. Two studies (10.0%)^(21,26)^ reported contamination of privacy curtains between beds, indicating hand contact by healthcare professionals as the likely route of cross-contamination. The characteristics of the contaminated fabrics, including their intended use, the types of microorganisms identified, the presence of multidrug-resistant organisms, and strategies developed for the treatment and processing of textiles aimed at eradicating outbreaks and preventing contamination are outlined in Chart 2. A reduction in HCAIs rates was observed with the use of copper oxide-treated fabrics, particularly sheets used in acute and critical care units^(33,34,36,37)^. This is summarized in Supplement 3^(18)^. These studies considered the type of fabric, the patient population, and HCAIs incidence rates before and after the application of biocidal technologies.

### Textile Characteristics, Identified Microorganisms, and Sources of Contamination

Among the studies that associated textile contamination with the occurrence of HCAIs, the microorganisms found were predominantly bacteria (n = 11; 55%) and fungi (n = 3; 15%). The most frequently reported pathogen was the bacterium *Bacillus cereus* (n = 7; 35%)^(20,22,23,24,28,29,35)^, followed by isolated mentions of methicillin-resistant *Staphylococcus aureus* (MRSA) (n = 1; 5%)^(32)^, Group A *Streptococcus* (n = 1; 5%)^(26)^, *Klebsiella pneumoniae* ESBL (n = 1; 5%)^(30)^, and *Acinetobacter baumannii* (n = 1; 5%)^(21)^. Among the fungi, *Rhizopus* was highlighted in three studies (15.0%)^(25,27,29)^ (Chart 2).

The studies included in this review describe a range of practices for the safe use of textiles to minimize contamination and contribute to the prevention of HCAIs. These practices include appropriate handling, processing, storage, and transport of textiles, the safety of laundering equipment, and surveillance and control measures, among other strategies (Chart 3).

**Chart 3:** Handling, processing, storage, transportation, equipment maintenance and surveillance strategies identified in the included studies – Caraguatatuba, SP, Brazil, 2025.

Manipulation		
Actions	Strategy/Motive	Studies
During the preparation of beds and the replacement of patients’ garments.	Avoid movements that may promote the dispersion of contaminant particles suspended in the air.	^(32)^
Following bed preparation.	Disinfect the surfaces in the environment after bed preparation, which may involve the dispersion of suspended particles, alongside handwashing by the professional.	^(32)^
Handwashing and the use of appropriate Personal Protective Equipment (PPE) when handling textiles throughout the entire process.	Good practice in hand hygiene and the use of PPE should be followed as a standard control protocol when handling textiles by the cleaning, care, and hospitality teams.	^(23,30)^
Attention should be given when handling clothing in environments that pose risks.	Cross-contamination between clean and soiled materials should be avoided, as well as exposing laundered clothing to unsanitary environments.	^(25)^
**Processing (laundering)**		
Establish laundering protocols.	Laundries should have specific protocols regarding time, temperature, and sanitizers for textile hygiene.	^(26,28)^
Consider special care for textiles used in environments and patients colonized by multidrug-resistant microorganisms.	The need for the use of specific substances in the washing of textiles used by patients colonized by microorganisms relevant to the etiology of HCAIs should be considered.	^(22)^
Pay attention to the presence of stains or visible dirt.	Items should be segregated and prepared in a manner that maximizes the effectiveness of cleaning for each piece, as the presence of stains or visible dirt may indicate a higher likelihood of contamination.	^(30)^
**Storage and transportation**		
Attention to the humidity and temperature in storage areas.	Attention to temperature and humidity variations related to the seasons and the location where clean textiles are stored. High temperatures and humidity levels can favor microbial proliferation.	^(28)^
Ensure complete drying of textiles before storage.	Fabrics should be completely dry before the packaging and storage process. High temperatures and humidity levels can favor microbial proliferation.	^(35)^
Strategy for the use of bed linens based on storage time.	Control should be maintained over the storage period of textiles, ensuring that items stored first are used first.	^(35)^
Quality of transport equipment and storage furniture.	The equipment used for the transport and storage of textiles should be properly cleaned/decontaminated and assessed for integrity. Cabinets should be evaluated for the presence of mold and the integrity of doors, as well as for exposure to residues and airborne particles.	^(25,29)^
**Maintenance of Equipment**		
Checking the functionality of equipment.	Regularly check the functionality and calibration of the sensors on washing and drying machines to ensure they operate at the desired temperature. There is a risk of biofilm formation or the development of resistant strains of microorganisms when pathogenic microorganisms are not sufficiently eradicated during the cycles.	^(20,27,30,35)^
**Control surveillance**		
Strict control of healthcare-associated infection (HAI) indicators in the units served by the laundry.	Verify with the care units the indicators of HCAIs and any potential association with an increase in cases related to contaminated textiles.	^(28)^
Laundry audits.	The laundry process and washing cycles should be regularly reviewed and audited.	^(27,35)^
Surveillance cultures of textiles.	Regular surveillance cultures should be performed to assess an acceptable quantity of potentially pathogenic microorganisms. Routine microbiological monitoring in laundries serves as a quality control measure and helps minimize risks related to hospital pathogens that form heat-resistant spores.	^(19,20,24,28,38)^
Attention to the surveillance of immunocompromised patients.	Quality control surveillance and regular replacement of fabrics in direct contact with the skin of immunocompromised patients should be ensured.	^(24)^
**Other strategies**		
Replacement of textiles with other materials that are easier to sanitize when appropriate.	Curtains and other textiles commonly used, such as screens, should be replaced with materials that are easy to clean. When replacement is not possible, a control system for the frequency of changes and washing should be maintained, as fabric contamination is inevitable.	^(21,26,30)^
Use of technologies.	Biocidal fabric compositions as an alternative for reducing healthcare-associated infection (HAI) rates.	^(32,33,34,36,37)^
Define specific protocols that meet the institutional profile and demand.	Define institutional protocols tailored to each specific context.	^(31)^

## DISCUSSION

This review identified that bacteria, particularly *Bacillus cereus*, were the most prevalent microorganisms associated with contamination of textiles in contact with patients’ skin. These contaminants were linked to infections of various natures, both fatal and non-fatal, affecting from newborns to the elderly in diverse healthcare settings. The cases of contamination were primarily related to handling, cleaning, processing, and storage practices. There was a limited body of research concerning the use of copper oxide in the manufacture of fabrics, aimed at preventing HCAIs.

Several studies included in this review investigated outbreaks associated with *B. cereus* as a contaminant of textiles, frequently identified in hospital settings. *B. cereus* is a bacterium of significant concern in food safety, known for its high resistance to conventional sterilization methods due to its ability to produce spores, which protect it against adverse conditions^(39)^. Notably, *B. cereus* exhibits resistance to povidone-iodine and alcohol, and sterilization using hot water or steam at 100°C is ineffective. Effective eradication methods include heat treatments above 160°C and the use of high-level disinfectants^(28)^.

The bacterium *B. cereus* can replicate and sporulate on dirty, moist fabric at high temperatures, making it challenging to remove even with effective washing processes. Since spores are not inactivated by thermal disinfection, removal through dilution is necessary^(24)^. Consequently, contamination occurs when dirty fabric enters the washing machine, spreading contamination to other fabrics and equipment involved in the washing process^(35)^. Furthermore, *B. cereus* is capable of forming biofilms, which complicates its elimination from hospital surfaces and promotes its persistence in environments such as central venous catheters, where it has been associated with cases of bacteremia, particularly in immunosuppressed patients^(40)^.

By recognizing the potential contamination of textiles and the primary microorganisms involved, healthcare institutions can develop protocols that are tailored to their specific circumstances. International guidelines recommend adequate sanitation of hospital linens, which involves a washing process at a minimum temperature of 71°C for 25 minutes, or alternatively, the use of effective chemical products for decontamination. Temperature plays a crucial role in the textile sanitation process, as it reduces the surface tension of water, improving its penetration into fabric fibers and aiding in the removal of contaminants. However, it is important to note that inadequate temperatures during washing can promote the persistence of resistant microorganisms, which require specific control measures, as observed in the studies included in this review regarding *B. cereus* ^(30)^.

Certainly, microbial contamination is not the only issue arising from ineffective textile processing, as efficient rinsing is essential to remove chemical residues and prevent potential skin irritation both in patients and healthcare professionals. Furthermore, microbiological control of hospital linen must be regularly monitored, and the equipment used in the washing process must undergo periodic maintenance to prevent failures that could compromise disinfection^(20,27,30,35)^.

It is also worth highlighting that the occurrence of HCAIs outbreaks has shown a seasonal pattern in several study contexts, with higher frequencies observed during the summer or warmer months^(22,24,29)^. The combination of heat and humidity creates conditions conducive to microbial growth, increasing the risk of contamination in hospital environments. When coupled with inadequate hygiene protocols for fabrics, such as uniforms and bed linen, this can contribute to the spread of various pathogens, including fungi from the genus *Rhizopus* ^(29)^.

It was observed that the studies included in this review emphasized the need to establish protocols and strategies for handling textiles and conducting surveillance cultures as essential measures to prevent significant contamination. The guidelines outlined in these studies were either based on or closely aligned with the recommendations from authoritative sources such as the 2003 *Guidelines for Environmental Infection Control in Health-Care Facilities* by the Centers for Disease Control and Prevention (CDC) and the Healthcare Infection Control Practices Advisory Committee (HICPAC)^(41)^. These guidelines provide instructions for the safe handling of soiled linen, aiming to minimize the dispersion of contaminants.

Among these recommendations, it is important to highlight that both internal and external hospital laundries must adhere to strict hygiene, transportation, and microbiological control standards to prevent cross-contamination. For outsourced services, it is essential for the hospital to conduct regular audits and inspections to ensure compliance with these guidelines. Additionally, all laundries must provide training for employees on the proper use of personal protective equipment (PPE) and biosafety protocols, ensuring that hospital linens are processed safely and effectively^(42)^.

Another aspect that should be considered is the contamination of textiles not directly involved in patient care but still subject to high levels of contamination, such as lab coats, curtains, and screens. Research on the physical barrier function of polyester fabric against the passage of fluids and bacteria in nursing professionals’ lab coats highlights the importance of improving manufacturing techniques for fabrics with biocidal technology. This is supported by studies reporting significant reductions in HCAIs rates in the services where these fabrics were tested^(43)^. In these instances, the use of technologies, such as the incorporation of biocidal agents into fabric manufacture, was associated with a reduction in antibiotic consumption and the duration of febrile episodes. Furthermore, sheets containing copper oxide have been shown to contribute to significant cost savings related to hospitalization duration and antibiotic use^(33)^.

Thus, developing strategies for textile quality control requires the establishment of rational approaches to minimize risks and optimize practices aimed at enhancing patient safety actions^(29,44)^. The key findings of this review underscore the importance of understanding the behavior of relevant microorganisms in textile contamination across different geographic regions, taking into account the local microbiome and climatic variables, such as temperature and humidity. Additionally, the review highlights the significance of identifying methods and techniques for handling and sanitizing textiles, addressing parameters such as temperature, exposure time, and the most effective sanitizing agents for washing processes. The review also covers suitable resources and materials for storage and stockpiling, as well as the application of advanced technologies in fabric manufacture, with the goal of reducing microbial loads in fabrics used within the healthcare context.

Although there are similar reviews on the use of textiles as potential sources of contamination, these reviews focused solely on studies addressing fabric contamination, without considering textile contamination associated with HCAIs. Another limitation was the absence of methodological details, including the strategy used to select potentially eligible studies, as well as the descriptors and databases employed^(15,45)^.

## CONCLUSION

The relationship between the handling, processing, and storage of textiles is directly linked to the presence of contamination by pathogenic microorganisms and plays a pivotal role in the occurrence of HCAIs in hospital environments. Inadequate handling, processing, and storage of these materials can facilitate the spread of microorganisms, compromising the safety of both patients and healthcare professionals. Furthermore, inadequate handling and continuous exposure to contaminated surfaces increase the risk of transmission, underscoring the need for strict protocols for managing textile materials in healthcare settings, tailored to the microbiological profile detected.

In this context, ongoing surveillance of HAIs is crucial for identifying deficiencies in cleaning processes, assessing the effectiveness of adopted protocols, and implementing corrective actions to reduce the incidence of infections. Therefore, it is essential to implement control and prevention strategies that include continuous professional training, improvements in disinfection methods, and the adoption of more efficient technologies in the production and/or decontamination of hospital materials, with the option of replacing materials when deemed necessary.

Strict control of equipment used in the washing and processing of hospital textiles is a critical factor in ensuring effective decontamination. This requires periodic maintenance, calibration, and microbiological monitoring to prevent operational failures. Additionally, proper storage of these materials is equally important, as contact with contaminated surfaces after washing can compromise the entire cleaning process. Therefore, appropriate storage must adhere to specific biosafety standards to ensure that the fabrics are protected from recontamination before use.

Controlling HAIs requires an institutional commitment to active surveillance, the quality of hospital processes, and adherence to strict protocols, ensuring that hospital textiles are handled, processed, and stored safely. This will ultimately reduce the negative impacts on patient health and improve the overall effectiveness of healthcare services.

## Data Availability

https://doi.org/10.48331/SCIELODATA.6DMHMC
