# Supplementary material for: Manipulation and treatment of textiles and their relationship with healthcare-associated infections: a scoping review
Source: Rev Esc Enferm USP. 2026 Apr 27;60:e20250430. doi: 10.1590/1980-220X-REEUSP-2025-0430en (PMC13120689; doi:10.1590/1980-220X-REEUSP-2025-0430en)
Supplement: Translation

# Translation: Manipulación y tratamiento de textiles y su relación con las infecciones asociadas a la atención médica: una revisión del alcance

**DOI:** 10.1590/1980-220X-REEUSP-2025-0430es

**Authors:** Talita Tavares Della Motta, Adriane Reis Barletta Canicoba, Alessandra Yuri Takehana de Andrade, Ramon Antônio Oliveira, Renata Cristina de Campos Pereira Silveira, Vanessa de Brito Poveda

**Affiliations:** 1 Universidade de São Paulo, Escola de Enfermagem, Departamento de Enfermagem Médico-cirúrgica, São Paulo, SP, Brasil.; 2 Universidade de São Paulo, Escola de Enfermagem de Ribeirão Preto, Departamento de Enfermagem Fundamental, Ribeirão Preto, SP, Brasil.

**Keywords:** Textiles, Infección Hospitalaria, Ropa de Cama y Ropa Blanca, Vestuario, Control de Infecciones

## Abstract

**Background:**

Los textiles son materiales esenciales e indispensables en la atención sanitaria, y abarcan una amplia gama de artículos como ropa para pacientes, uniformes para el personal, ropa de cama, cortinas y campos estériles reutilizables. Sin embargo, estos materiales pueden convertirse en vehículos de contaminación, contribuir a la propagación de microorganismos o incluso aumentar las tasas de infecciones asociadas a la atención sanitaria (IAAS).

**Objetivo:**

Mapear la evidencia disponible en la literatura científica respecto al manejo, procesamiento y contaminación de textiles y la ocurrencia de IAAS.

**Método:**

Se realizó una revisión del alcance siguiendo las recomendaciones del JBI. Se consultaron las siguientes bases de datos: Web of Science, PubMed/Medline, CINAHL, LILACS, EMBASE, SCOPUS y SCIELO. La literatura gris se buscó a través del Catálogo de Tesis y Disertaciones de CAPES, MEDNAR y Google Scholar. La eliminación de duplicados, la selección independiente, la extracción de datos y la síntesis fueron realizadas por dos revisores, y cualquier discrepancia fue resuelta por un tercer evaluador. Se incluyeron estudios observacionales o experimentales, publicados en cualquier idioma y sin restricción de tiempo. Los datos se presentaron mediante síntesis descriptiva y tablas conteniendo información relevante.

**Resultados:**

Se incluyeron veinte estudios, siendo las series de casos el diseño de estudio más común (14; 70%). El tema principal fue la investigación de brotes, con *Bacillus cereus* (7; 35,0%) emergiendo como el microorganismo más significativo contaminante de los textiles que entran en contacto con la piel de los pacientes. La contaminación estaba relacionada con las técnicas de manipulación, limpieza, procesamiento y prácticas de almacenamiento. Se sugirió el uso de óxido de cobre como una estrategia en la producción de tejidos destinada a reducir la contaminación y las tasas de IAAS.

**Conclusión:**

El control de la contaminación textil requiere un compromiso institucional con la vigilancia activa, procesos hospitalarios de calidad y adhesión a protocolos estrictos, garantizando un manejo, procesamiento y almacenamiento seguros, reduciendo así los impactos negativos en la salud del paciente y la eficacia de los servicios prestados.

## INTRODUCCIÓN

Las infecciones asociadas a la atención de la salud (IAAS) se encuentran entre los eventos adversos más frecuentes en los entornos hospitalarios de todo el mundo y tienen repercusiones significativas en el estado clínico, emocional y funcional del paciente, además de generar impactos económicos en las instituciones y los sistemas de salud^(1)^.

Según la Organización Mundial de la Salud (OMS), aproximadamente 22 de cada 100 pacientes ingresados en hospitales de cuidados agudos contraen al menos una IAAS, y los pacientes de países de ingresos bajos y medios presentan casi el doble de casos que los de países de ingresos altos (15 frente a 7 pacientes afectados por cada 100 ingresos, respectivamente)^(2)^.

La etiología de las IAAS es multifactorial e implica factores intrínsecos del paciente y aspectos relacionados con los procesos de atención, como las condiciones ambientales y las características de los materiales y dispositivos utilizados^(3,4)^.

Los textiles, como la ropa de cama, los uniformes, las cortinas y los paños estériles reutilizables, son componentes esenciales de los entornos sanitarios y están en contacto directo tanto con los pacientes como con los profesionales de la salud.

Si bien estos materiales se utilizan para el cuidado y la protección de los pacientes y el personal, en ocasiones pueden servir como vectores de contaminación y contribuir a la diseminación de microorganismos, lo que en última instancia aumenta las tasas de IAAS. Esto se debe principalmente a la naturaleza porosa de las fibras textiles, que facilita el crecimiento microbiano, promueve la formación de biopelículas y permite la contaminación de otras superficies por contacto^(5,6,7)^.

Incluso los textiles que no entran en contacto directo con el cuerpo de los pacientes o los profesionales sanitarios, como las cortinas de privacidad entre las camas de los hospitales, requieren un cuidado especial y frecuente para garantizar una descontaminación rutinaria adecuada en las instituciones donde se utilizan. Estos artículos suelen albergar una carga microbiana considerable, incluidos microorganismos multirresistentes que desempeñan un papel importante en la patogénesis de las IAAS^(8)^.

En este contexto, los textiles hospitalarios pueden actuar como reservorios potenciales de microorganismos asociados a las IAAS, especialmente en áreas críticas como las unidades de cuidados intensivos, los servicios de urgencias y las salas de cirugía, albergando patógenos clínicamente relevantes como *Staphylococcus aureus* (incluido el *Staphylococcus aureus resistente a la meticilina (SARM)*), *Escherichia coli*, *Pseudomonas aeruginosa*, *Clostridioides difficile* y *Acinetobacter baumannii*, que son capaces de sobrevivir durante períodos prolongados en los tejidos, variando su comportamiento según el tipo de fibra, la humedad y las condiciones ambientales^(9)^.

Por lo tanto, deben tenerse en cuenta las características del tejido, la adhesión de microorganismos patógenos al tejido, la técnica de limpieza requerida para cada tejido según su composición y el tipo de contacto con la piel de los usuarios de los servicios de salud. En este contexto, un estudio que analizó la adhesión y la persistencia de las bacterias en los textiles hospitalarios demostró que microorganismos como las bacterias y los hongos pueden sobrevivir durante aproximadamente 26 días en diversos tipos de materiales, entre ellos el algodón, el polipropileno, el poliéster, la viscosa, la seda y las fibras de lana. Además, la duración de la persistencia microbiana y la carga bacteriana presente en las fibras textiles se asociaron con las características fisicoquímicas, la nanorrugosidad del tejido y las propiedades de la superficie que influyen en la capacidad de adhesión de cada microorganismo a la fibra^(10)^.

Un estudio in vitro realizado con piel porcina, que tiene características similares a la piel humana, demostró que es posible transferir microorganismos multirresistentes desde la superficie seca de un textil a la piel del animal^(11)^. Los resultados de otra investigación de laboratorio indicaron un aumento de las tasas de contaminación de los tejidos cuando los microorganismos se habían inoculado recientemente en el textil, siendo la transferencia cinco veces mayor cuando el tejido estaba húmedo o cuando se frotaba contra la piel^(12)^. A la luz de estos hallazgos, se han desarrollado tecnologías para la producción y el tratamiento de textiles, como tejidos impregnados con agentes antimicrobianos que han demostrado su eficacia para controlar la contaminación, reducir la carga microbiana y disminuir la incidencia de IAAS. No obstante, siguen existiendo importantes lagunas, como la escasez de datos sobre el comportamiento de las comunidades microbianas en los textiles utilizados en entornos sanitarios reales, así como el potencial de resistencia a los antimicrobianos y citotoxicidad^(5,9,13,14,15)^.

Así, en febrero de 2025, se realizaron búsquedas de posibles revisiones anteriores en las bases de datos electrónicas EPISTEMONIKOS, Cochrane Library, Open Science Framework y PubMed/Medline, y no se encontraron registros de revisiones que utilizaran únicamente estudios que asociaran la presencia de textiles contaminados con las tasas de IAAS^(7,10)^.

Por lo tanto, dada la ausencia de revisiones relevantes sobre el tema, a pesar de la importancia potencial de los textiles como probables vehículos de contaminación en los servicios sanitarios, es obligatorio identificar la evidencia existente, su calidad y las posibles lagunas relacionadas con los textiles como fómites en la etiología de las IAAS, los factores que contribuyen a la contaminación de estos materiales y las estrategias adecuadas de manipulación e higiene. En este sentido, los resultados del presente estudio pueden apoyar la toma de decisiones administrativas, mejorar las prácticas de los equipos de limpieza y enfermería y fortalecer las estrategias de los servicios de control de infecciones.

### Objetivo

Mapear la evidencia disponible en la literatura científica sobre la relación entre la manipulación y el procesamiento de materiales textiles, la presencia de contaminación por microorganismos patógenos y la aparición de IAAS en entornos hospitalarios.

## MÉTODO

Se trata de una revisión de alcance, que sigue las recomendaciones del Manual JBI para la síntesis de evidencia y se presenta de acuerdo con los Elementos de información preferidos para las revisiones sistemáticas y los metaanálisis de revisión de alcance (PRISMA-Scr)^(16)^. La pregunta de investigación se formuló basándose en el acrónimo PCC (P: población de interés; C: concepto; C: contexto), que se refiere, respectivamente, a los textiles utilizados en la atención sanitaria («Población»), la contaminación y la aparición de IAAS («Concepto») y los entornos de atención sanitaria hospitalaria («Contexto») descritos en el Cuadro 1. Por lo tanto, la pregunta que aborda esta revisión es: ¿Cuál es la evidencia sobre la relación entre la manipulación y el procesamiento de los textiles y su contaminación, y la aparición de IAAS en entornos hospitalarios? El protocolo se registró en la plataforma Open Science Framework (OSF), DOI https://doi.org/10.17605/OSF.IO/GNC8M.

**Cuadro 1:** Criterios PCC de inclusión y exclusión – Caraguatatuba, SP, Brasil, 2025.

	Sigla PCC	Criterios de inclusión	Criterios de exclusión
P	“Población”	Textiles utilizados en el sector de la salud	Tela no tejida (NWF por sus siglas en inglés) o materiales desechables
C	“Concepto”	Se incluyeron estudios que abordaban la contaminación textil, los métodos de manipulación y tratamiento, y la aparición de IAAS en entornos hospitalarios.	Los que abordan la contaminación de los textiles sin una asociación específica con las IAAS.
C	“Contexto”	Entornos de atención de salud hospitalarios	Estudios realizados en contextos no hospitalarios (como odontología o medicina veterinaria)

Se incluyeron estudios primarios cuantitativos, sin limitaciones en cuanto al idioma o el período de publicación, incluyendo informes de casos, series de casos, estudios transversales, estudios de cohortes, estudios de casos y controles, estudios antes y después, estudios experimentales o cuasi-experimentales y guías clínicas. Además, se incluyeron estudios que abordaban la contaminación textil, los métodos de manipulación y tratamiento, y la aparición de IAAS en entornos hospitalarios. Se excluyeron los estudios que se centraban exclusivamente en el uso de telas no tejidas (NWF) o materiales desechables, los que abordaban la contaminación de los textiles sin una asociación específica con las IAAS, los estudios realizados en contextos no hospitalarios (como la odontología o la medicina veterinaria), los estudios con animales, los estudios in vitro, las cartas al editor, los ensayos teóricos y los resúmenes presentados en conferencias.

### Recolección de Pruebas

Los términos o descriptores utilizados para la investigación en las bases de datos indexadas se seleccionaron de los Medical Subject Headings (MeSH) y los Health Sciences Descriptors (DeCS), utilizando una combinación de descriptores controlados y no controlados para garantizar una búsqueda exhaustiva, con la ayuda de un bibliotecario especializado^(17)^, (Suplemento 1)^(18)^.

La búsqueda de estudios se llevó a cabo en abril y mayo de 2024 en las siguientes bases de datos: Web of Science, PubMed/Medline, Índice Acumulativo de Literatura de Enfermería y Salud Aliada (CINAHL), Literatura Latinoamericana y del Caribe en Ciencias de la Salud (LiLACS), EMBASE, SCOPUS y Biblioteca Científica Electrónica en Línea (SciELO), sin restricciones de tiempo ni de idioma. La búsqueda en la literatura gris se realizó en julio de 2024 en los catálogos de tesis y disertaciones de CAPES y MEDNAR, así como en Google Scholar, revisando todos los registros. Por último, también se revisaron las referencias de los estudios seleccionados para identificar estudios relevantes.

### Selección de los Registros Recuperados

Los resultados recuperados de las bases de datos se importaron a la plataforma Rayyan Intelligent Systematic Reviews. Tras eliminar los duplicados, se seleccionaron los títulos y resúmenes de acuerdo con los criterios de inclusión y exclusión predefinidos, sin utilizar herramientas de inteligencia artificial. Posteriormente, se obtuvieron los artículos completos de los estudios seleccionados y dos revisores los evaluaron de forma independiente, utilizando la función de cegamiento de la plataforma. Cualquier desacuerdo durante el proceso de selección fue resuelto por un tercer revisor.

### Síntesis de Datos

Los datos fueron extraídos de forma independiente por dos revisores. En caso de incertidumbre o desacuerdo, se consultó a un tercer experto para llegar a un consenso. La síntesis de los datos se realizó de forma descriptiva y se compilaron tablas narrativas para presentar la información relevante de manera estructurada.

## RESULTADOS

Se recuperaron un total de 4447 registros de las bases de datos evaluadas. Tras eliminar los duplicados, quedaron 4099 manuscritos para la selección de títulos y resúmenes. De estos, 108 artículos se sometieron a una evaluación del texto completo, lo que dio como resultado la selección de 16 estudios para su inclusión en la revisión. Al mismo tiempo, se identificaron 482 registros potencialmente elegibles a través de fuentes de literatura gris y cuatro cumplieron los criterios de inclusión. Así, la muestra final comprendió 20 estudios, que se incluyeron en esta revisión de alcance (Figura 1).

**Figura 1:** Flujograma de seleción de estudios adaptada de PRISMA – ScR – Caraguatatuba, SP Brasil,

### Características Generales de los Estudios Incluidos

La muestra final comprendió 20 estudios publicados entre 1981 y 2020, con un diseño de serie de casos empleado en 12^(19,20,21,22,23,24,25,26,27,28,29,30)^. La mayoría de los estudios se publicaron en inglés (n = 18; 90%)^(19,20,21,22,23,24,25,26,27,29,30,31,32,33,34,35,36,37)^, uno en italiano (5%)^(38)^ y otro en japonés (5%)^(28)^. Los países con mayor número de publicaciones sobre el tema fueron Estados Unidos (n = 5; 25%)^(25,29,31,34,37)^, Reino Unido (n = 5; 25%)^(19,20,21,24,26)^, Japón (n = 4; 20%)^(22,23,28,32)^, China (n = 2; 10%)^(27,35)^ e Israel (n = 2; 10%)^(33,36)^, seguidos de los Países Bajos (n = 1; 5%)^(30)^ e Italia (n = 1; 5%)^(38)^ (Suplemento 2)^(18)^.

En cuanto al tema principal, la mayoría de los estudios (n = 14; 70,0%)^(19,20,21,22,23,24,25,26,27,28,29,30,32,35)^ investigaron la relación entre la presencia de textiles contaminados por microorganismos y la aparición de IAAS en unidades sanitarias. Cuatro estudios (20,0%)^(28,32,35,36)^ abordaron el uso de compuestos biocidas, como el óxido de cobre, en la fabricación de tejidos como estrategia para reducir la contaminación y las tasas de IAAS. Otros dos estudios (10,0%) exploraron otras estrategias relacionadas con los textiles, incluida la evaluación del impacto del uso de batas privadas en las tasas de IAAS en una unidad de cuidados intensivos neonatales^(31)^ y una comparación de las batas y paños de tela reutilizables con los paños desechables en relación con las tasas de infección del sitio quirúrgico^(38)^(Suplemento 2)^(18)^.

Algunos estudios (n = 3; 15%)^(22,24,29)^ informaron de brotes estacionales de infección relacionados con las variaciones climáticas y de temperatura, especialmente durante los meses más cálidos del año, que favorecen la proliferación y la supervivencia de microorganismos que se desarrollan en condiciones de calor y humedad. Además, la contaminación observada en los textiles se asoció con una serie de resultados, entre los que destacan la bacteriemia, las infecciones cutáneas y las infecciones respiratorias, que dieron lugar a eventos adversos mortales o no mortales (Cuadro 2).

**Cuadro 2:** Tipo de tejido, microorganismos identificados, perfil de resistencia, origen de la contaminación y estrategia de control – Caraguatatuba, SP, Brasil, 2025.

Estudio	Tipo de microorganismo (Microorganismo)	Presencia de multirresistencia (antibióticos)	Tipo de tejido contaminado	Tejido en contacto con la piel	Origen de la contaminación	Estrategia utilizada/sugerida para el tratamiento/procesamiento de textiles
Birch *et al*. [18]	Bacteria *(B. cereus)*	NI	Pañales limpios	Sí	Contaminación de la lavadora debido al uso de almidón de arroz	Control de los equipos de lavado de tejidos y cultivos de vigilancia posteriores al lavado.
Barrie *et al*. [19]	Bacteria *(B. cereus)*	NI	Ropa de cama	Sí	Ropa de cama utilizada y almacenada húmeda en bolsas de plástico, a altas temperaturas ambientales	Desinfección de la lavadora con hipoclorito durante 18 horas y vapor (temperatura de 82 °C). Control microbiológico rutinario.
Das *et al*. [22]	Bacteria *(A. baumanii)*	Sí (Carbapenem)	Cortina de privacidad	No	Probable contaminación por las manos	Aumento de la frecuencia de limpieza del entorno con hipoclorito (1000 ppm). Las cortinas se cambian dos veces por semana. Uso restringido de meropenem en las unidades. Higiene de manos con agua y jabón o clorhexidina acuosa O alcohol. Recordatorios para usar guantes y delantales en el contacto directo con pacientes con riesgo de transmisión.
Shiomori, *et al*. [23]	Bacteria *(MRSA)*	Sí (meticilina)	Ropa de cama	Sí	Dispersión de microorganismos durante el cambio de sábanas contaminadas	Formación continua sobre la limpieza de camas para todo el personal médico y de enfermería.
Dohmae *et al*. [24]	Bacteria *(B. cereus)*	Sí (Ciprofloxacina/eritromicina/tetraciclina/gentamicina/minociclina)	Toallas	Sí	Proceso de desinfección ineficaz	Uso de sustancias específicas en el lavado de tejidos utilizados por pacientes colonizados por microorganismos patógenos relevantes, como el uso de hipoclorito de sodio para eliminar la bacteria *B. cereus*.
Sasahara *et al.* [25]	Bacterium *(B. cereus)*	NI	Ropa de cama: sábanas y toallas	Sí	Contaminación de la lavadora debido al uso de agua reciclada y al mantenimiento deficiente	Sábanas hospitalarias esterilizadas en autoclave. Desinfección de la lavadora con un potente detergente alcalino. Suspensión del reciclaje de agua para el lavado y el aclarado. Además, se exigió al personal hospitalario que utilizara guantes al manipular dispositivos intravenosos, no solo durante la punción, sino también durante la administración de la terapia intravenosa.
Hosein *et al*. [26]	Bacteria (*B. cereus*)	NI	Ropa de cama de la cuna: manta, sábana, funda de almohada	Sí	Proceso de saneamiento ineficaz	Aumento del caudal de agua. Monitorización microbiológica rutinaria.
Duffy *et al.* [27]	Hongo (*Rhizopus delemar*)	NI	Ropa de cama: sábanas, mantas, fundas de almohada, toallas y batas de paciente	Sí	Contaminación en la lavandería o durante la entrega	Cambio en la empresa proveedora de ropa de cama. Cambios estructurales en la entrega. Eliminación de la ropa de cama antigua del hospital. Limpieza y desinfección de las zonas de almacenamiento de la ropa de cama. Uso temporal de ropa de cama esterilizada en autoclave para pacientes considerados de alto riesgo de infección.
Mahida *et al.* [29]	Bacterias (*estreptococo A)*	NI	Cortinas de privacidad entre camas	No	Contaminación cruzada por contacto con las manos de los profesionales	Sustituir las cortinas por materiales fáciles de limpiar. Lleve un registro de la frecuencia con la que cambia y lava las cortinas.
Cheng *et al.* [30]	Hongos *(Rhizopus microsporus Zigomicetos)*	NI	Ropa de cama	Sí	Mal estado de la ropa – Suciedad en los ventiladores de pared, las salidas de los conductos de aire del techo y la superficie de las planchas – Almacenamiento: sábanas empaquetadas húmedas y calientes	Control microbiológico rutinario. Limpieza y desinfección de la unidad y los vehículos de reparto, incluidas las superficies y los equipos. Mantenimiento de las lavadoras (calibración de los sensores de temperatura, control de la humedad durante los procesos de secado y embalaje). Cambios estructurales: separación clara entre las zonas limpias y sucias para evitar la contaminación cruzada de los artículos procesados. Comprobación de las condiciones de almacenamiento de la ropa de cama (temperatura y humedad). Cambios rutinarios: consumo de ropa de cama según el principio «primero en entrar, primero en salir». Los médicos deben mantener un alto índice de sospecha para el diagnóstico y tratamiento tempranos de la zigomicosis en pacientes inmunodeprimidos.
Itoga *et al*. [31]	Bacteria *(B. cereus)*	NI	Toallas	Sí	Proceso de desinfección ineficaz	Procesos de tratamiento térmico por encima de 160 °C, uso de desinfectantes de alto nivel (ácido peracético e hipoclorito de sodio) durante el lavado. Control microbiológico durante la recepción de la ropa/al final de los procesos de higiene.
Teal *et al*. [33]	Hongos (*Rhizopus*)	NI	Ropa de cama	Sí	Contaminación de los carros de transporte de ropa de cama	Implementación de una rutina para vaciar y limpiar los carros de cambio de ropa de cama con detergente desinfectante registrado por la Agencia de Protección Ambiental (EPA). Cultivos de vigilancia. Cambio de la empresa proveedora de ropa de cama.
Cheng *et al.* [34]	Bacteria (*B. cereus)*	NI	Ropa de cama	Sí	Inhalación directa y contacto de la piel con artículos contaminados	Cambio de empresa proveedora de ropa de cama. Secado completo de la tela antes de guardarla.
Boonstra *et al*. [37]	Bacteria (*K. pneumoniae – ESBL*)	Sí (cefotaxima y/o ceftazidima, tobramicina, amikacina, kanamicina, ciprofloxacina y norfloxacina)	Ropa de cama	Sí	Contaminación de la lavadora debido a un uso inadecuado	Aislamiento de pacientes contaminados. Funcionamiento adecuado de la temperatura de la máquina durante el proceso de lavado.

La mayoría de los textiles contaminados eran artículos en contacto directo con la piel de los pacientes, como ropa de cama, sábanas, fundas de almohada, mantas, pañales y toallas de baño (n = 13; 65%)^(19,20,21,22,23,24,25,27,28,29,30,32,35)^. Dos estudios (10,0%)^(21,26)^ informaron de la contaminación de las cortinas de privacidad entre las camas, lo que indica que el contacto con las manos por parte del personal sanitario es la vía más probable de contaminación cruzada. Las características de los tejidos contaminados, incluido su uso previsto, los tipos de microorganismos identificados, la presencia de organismos multirresistentes y las estrategias desarrolladas para el tratamiento y procesamiento de los textiles con el fin de erradicar los brotes y prevenir la contaminación se describen en la Cuadro 2. Se observó una reducción de las tasas de IAAS con el uso de tejidos tratados con óxido de cobre, en particular sábanas utilizadas en unidades de cuidados intensivos y críticos^(33,34,36,37)^. Esto se resume en el suplemento 3^(18)^. Estos estudios tuvieron en cuenta el tipo de tejido, la población de pacientes y las tasas de incidencia de las IAAS antes y después de la aplicación de tecnologías biocidas.

### Características de los Textiles, Microorganismos Identificados y Fuentes de Contaminación

Entre los estudios que asociaron la contaminación de los textiles con la aparición de IAAS, los microorganismos encontrados fueron predominantemente bacterias (n = 11; 55%) y hongos (n = 3; 15%).

El patógeno más frecuentemente reportado fue la bacteria *Bacillus cereus* (n = 7; 35%)^(20,22,23,24,28,29,35)^, seguido de menciones aisladas de *Staphylococcus aureus* resistente a la meticilina (SARM) (n = 1; 5%)^(32)^, *Streptococcus* del grupo A (n = 1; 5%)^(26)^, *Klebsiella pneumoniae* ESBL (n = 1; 5%)^(30)^ y *Acinetobacter baumannii* (n = 1; 5%)^(21)^. Entre los hongos, *Rhizopus* se destacó en tres estudios (15,0%)^(25,27,29)^ (Cuadro 2).

Los estudios incluidos en esta revisión describen una serie de prácticas para el uso seguro de los textiles con el fin de minimizar la contaminación y contribuir a la prevención de las IAAS. Estas prácticas incluyen la manipulación, el procesamiento, el almacenamiento y el transporte adecuados de los textiles, la seguridad de los equipos de lavandería y las medidas de vigilancia y control, entre otras estrategias (Cuadro 3).

**Cuadro 3:** Estrategias de manipulación, procesamiento, almacenamiento, transporte, mantenimiento de equipos y vigilancia identificadas en los estudios incluidos – Caraguatatuba, SP, Brasil, 2025.

Manipulación		
Acciones	Estrategia/Motivo	Estudios
Durante la preparación de las camas y el cambio de ropa de los pacientes.	Evitar movimientos que puedan favorecer la dispersión de partículas contaminantes suspendidas en el aire.	^(32)^
Tras la preparación de la cama.	Desinfectar las superficies del entorno tras la preparación de la cama, lo que puede implicar la dispersión de partículas en suspensión, además del lavado de manos por parte del profesional.	^(32)^
Lavado de manos y uso de equipos de protección individual (EPI) adecuados durante la manipulación de textiles a lo largo de todo el proceso.	Se deben seguir las buenas prácticas en materia de higiene de las manos y el uso de EPI como protocolo de control estándar cuando los equipos de limpieza, cuidado y hostelería manipulen textiles.	^(23,30)^
Se debe prestar atención al manipular la ropa en entornos que presenten riesgos.	Debe evitarse la contaminación cruzada entre materiales limpios y sucios, así como exponer la ropa lavada a entornos insalubres.	^(25)^
**Procesamiento (lavado)**		
Establecer protocolos de lavado.	Las lavanderías deben contar con protocolos específicos en cuanto a tiempo, temperatura y desinfectantes para la higiene de los textiles.	^(26,28)^
Considerar un cuidado especial para los textiles utilizados en entornos y pacientes colonizados por microorganismos multirresistentes.	Se debe considerar la necesidad de utilizar sustancias específicas en el lavado de los textiles utilizados por pacientes colonizados por microorganismos relevantes para la etiología de las infecciones asociadas a la atención sanitaria.	^(22)^
Preste atención a la presencia de manchas o suciedad visible.	Los artículos deben separarse y prepararse de manera que se maximice la eficacia de la limpieza de cada pieza, ya que la presencia de manchas o suciedad visible puede indicar una mayor probabilidad de contaminación.	^(30)^
**Almacenamiento y transporte**		
Atención a la humedad y la temperatura en las zonas de almacenamiento.	Atención a las variaciones de temperatura y humedad relacionadas con las estaciones del año y la ubicación donde se almacenan los textiles limpios. Las altas temperaturas y los altos niveles de humedad pueden favorecer la proliferación microbiana.	^(28)^
Asegúrese de que los textiles estén completamente secos antes de almacenarlos.	Los tejidos deben estar completamente secos antes del proceso de embalaje y almacenamiento. Las altas temperaturas y los niveles de humedad pueden favorecer la proliferación microbiana.	^(35)^
Estrategia para el uso de la ropa de cama en función del tiempo de almacenamiento.	Se debe controlar el periodo de almacenamiento de los textiles, asegurándose de que los artículos almacenados primero sean los primeros en utilizarse.	^(35)^
Calidad del equipo de transporte y del mobiliario de almacenamiento.	El equipo utilizado para el transporte y almacenamiento de textiles debe limpiarse/descontaminarse adecuadamente y evaluarse para comprobar su integridad. Los armarios deben evaluarse para detectar la presencia de moho y la integridad de las puertas, así como la exposición a residuos y partículas en suspensión.	^(25,29)^
**Mantenimiento del equipo**		
Comprobación del funcionamiento del equipo.	Comprobar periódicamente el funcionamiento y la calibración de los sensores de las lavadoras y secadoras para garantizar que funcionan a la temperatura deseada. Existe el riesgo de formación de biopelículas o de desarrollo de cepas resistentes de microorganismos cuando los microorganismos patógenos no se erradican suficientemente durante los ciclos.	^(20,27,30,35)^
**Control de vigilancia**		
Control estricto de los indicadores de infecciones asociadas a la asistencia sanitaria (IAA) en las unidades atendidas por la lavandería.	Verificar con las unidades asistenciales los indicadores de IAAS y cualquier posible asociación con un aumento de los casos relacionados con textiles contaminados.	^(28)^
Auditorías de lavandería.	El proceso de lavandería y los ciclos de lavado deben revisarse y auditarse periódicamente.	^(27,35)^
Cultivos de vigilancia de textiles.	Se deben realizar cultivos de vigilancia periódicos para evaluar una cantidad aceptable de microorganismos potencialmente patógenos. El control microbiológico rutinario en las lavanderías sirve como medida de control de calidad y ayuda a minimizar los riesgos relacionados con los patógenos hospitalarios que forman esporas resistentes al calor.	^(19,20,24,28,38)^
Atención a la vigilancia de los pacientes inmunodeprimidos.	Se debe garantizar la vigilancia del control de calidad y la sustitución periódica de los tejidos que están en contacto directo con la piel de los pacientes inmunodeprimidos.	^(24)^
**Otras estrategias**		
Sustitución de textiles por otros materiales más fáciles de desinfectar cuando sea apropiado.	Las cortinas y otros textiles de uso común, como las pantallas, deben sustituirse por materiales fáciles de limpiar. Cuando no sea posible sustituirlos, se debe mantener un sistema de control de la frecuencia de los cambios y lavados, ya que la contaminación de los tejidos es inevitable.	^(21,26,30)^
Uso de tecnologías.	Composiciones de tejidos biocidas como alternativa para reducir las tasas de infecciones asociadas a la asistencia sanitaria (HAI).	^(32,33,34,36,37)^
Definir protocolos específicos que se ajusten al perfil y la demanda de la institución.	Definir protocolos institucionales adaptados a cada contexto específico.	^(31)^

## DISCUSIÓN

Esta revisión identificó que las bacterias, en particular *Bacillus cereus*, eran los microorganismos más prevalentes asociados con la contaminación de los textiles en contacto con la piel de los pacientes. Estos contaminantes se relacionaron con infecciones de diversa naturaleza, tanto mortales como no mortales, que afectaban desde recién nacidos hasta ancianos en diversos entornos sanitarios. Los casos de contaminación se relacionaron principalmente con las prácticas de manipulación, limpieza, procesamiento y almacenamiento. Existía un conjunto limitado de investigaciones sobre el uso del óxido de cobre en la fabricación de tejidos, con el objetivo de prevenir las IAAS. Varios estudios incluidos en esta revisión investigaron brotes asociados con B. cereus como contaminante de textiles, frecuentemente identificado en entornos hospitalarios. B. cereus es una bacteria muy preocupante para la seguridad alimentaria, conocida por su alta resistencia a los métodos de esterilización convencionales debido a su capacidad para producir esporas, que la protegen contra condiciones adversas^(39)^.

Cabe destacar que B. cereus muestra resistencia a la povidona yodada y al alcohol, y que la esterilización con agua caliente o vapor a 100 °C es ineficaz. Los métodos de erradicación eficaces incluyen tratamientos térmicos por encima de 160 °C y el uso de desinfectantes de alto nivel^(28)^.

La bacteria *B. cereus* puede replicarse y esporular en tejidos sucios y húmedos a altas temperaturas, lo que dificulta su eliminación incluso con procesos de lavado eficaces. Dado que las esporas no se inactivan con la desinfección térmica, es necesario eliminarlas mediante dilución^(24)^. En consecuencia, la contaminación se produce cuando los tejidos sucios entran en la lavadora, propagándose a otros tejidos y equipos que intervienen en el proceso de lavado^(35)^.

Además, B. cereus es capaz de formar biopelículas, lo que complica su eliminación de las superficies hospitalarias y favorece su persistencia en entornos como los catéteres venosos centrales, donde se ha asociado a casos de bacteriemia, especialmente en pacientes inmunodeprimidos^(40)^. Al reconocer la posible contaminación de los textiles y los principales microorganismos implicados, las instituciones sanitarias pueden desarrollar protocolos adaptados a sus circunstancias específicas.

Las directrices internacionales recomiendan una higienización adecuada de la ropa de cama hospitalaria, lo que implica un proceso de lavado a una temperatura mínima de 71 °C durante 25 minutos o, alternativamente, el uso de productos químicos eficaces para la descontaminación. La temperatura desempeña un papel crucial en el proceso de higienización de los textiles, ya que reduce la tensión superficial del agua, mejorando su penetración en las fibras del tejido y ayudando a eliminar los contaminantes. Sin embargo, es importante señalar que unas temperaturas inadecuadas durante el lavado pueden favorecer la persistencia de microorganismos resistentes, que requieren medidas de control específicas, como se observa en los estudios incluidos en esta revisión sobre *B. cereus* ^(30)^.

Sin duda, la contaminación microbiana no es el único problema que se deriva de un procesamiento ineficaz de los textiles, ya que un enjuague eficaz es esencial para eliminar los residuos químicos y prevenir posibles irritaciones cutáneas tanto en los pacientes como en los profesionales sanitarios. Además, el control microbiológico de la ropa de cama hospitalaria debe monitorearse periódicamente, y el equipo utilizado en el proceso de lavado debe someterse a un mantenimiento periódico para evitar fallos que puedan comprometer la desinfección^(20,27,30,35)^.

También cabe destacar que la aparición de brotes de IAAS ha mostrado un patrón estacional en varios contextos de estudio, con frecuencias más altas observadas durante el verano o los meses más cálidos^(22,24,29)^.

La combinación de calor y humedad crea condiciones propicias para el crecimiento microbiano, lo que aumenta el riesgo de contaminación en los entornos hospitalarios. Si a esto se suman protocolos de higiene inadecuados para los tejidos, como los uniformes y la ropa de cama, se puede contribuir a la propagación de diversos patógenos, incluidos los hongos del género Rhizopus^(29)^.

Se observó que los estudios incluidos en esta revisión hacían hincapié en la necesidad de establecer protocolos y estrategias para la manipulación de textiles y la realización de cultivos de vigilancia como medidas esenciales para prevenir una contaminación significativa. Las directrices esbozadas en estos estudios se basaban o se ajustaban estrechamente a las recomendaciones de fuentes autorizadas, como las Directrices para el control de infecciones ambientales en centros de atención de salud de 2003 de los Centros para el Control y la Prevención de Enfermedades (CDC) y el Comité Asesor sobre Prácticas de Control de Infecciones en la Atención de Salud (Healthcare Infection Control Practices Advisory Committee – HICPAC en la sigla en inglés)^(41)^. Estas directrices proporcionan instrucciones para la manipulación segura de la ropa sucia, con el objetivo de minimizar la dispersión de contaminantes.

Entre estas recomendaciones, es importante destacar que tanto las lavanderías internas como las externas de los hospitales deben cumplir estrictas normas de higiene, transporte y control microbiológico para evitar la contaminación cruzada. En el caso de los servicios subcontratados, es esencial que el hospital realice auditorías e inspecciones periódicas para garantizar el cumplimiento de estas directrices. Además, todas las lavanderías deben impartir formación a los empleados sobre el uso adecuado de los equipos de protección personal (EPP) y los protocolos de bioseguridad, garantizando que la ropa de cama del hospital se procese de forma segura y eficaz^(42)^.

Otro aspecto que debe tenerse en cuenta es la contaminación de los textiles que no intervienen directamente en la atención al paciente, pero que siguen estando sujetos a altos niveles de contaminación, como las batas de laboratorio, las cortinas y las pantallas. Las investigaciones sobre la función de barrera física del tejido de poliéster contra el paso de fluidos y bacterias en las batas de laboratorio de los profesionales de enfermería ponen de relieve la importancia de mejorar las técnicas de fabricación de tejidos con tecnología biocida. Esto se ve respaldado por estudios que informan de reducciones significativas en las tasas de IAAS en los servicios donde se probaron estos tejidos^(43)^. En estos casos, el uso de tecnologías, como la incorporación de agentes biocidas en la fabricación de tejidos, se asoció con una reducción del consumo de antibióticos y de la duración de los episodios febriles. Además, se ha demostrado que las sábanas que contienen óxido de cobre contribuyen a un ahorro significativo de costos relacionados con la duración de la hospitalización y el uso de antibióticos^(33)^.

Por lo tanto, el desarrollo de estrategias para el control de calidad de los textiles requiere el establecimiento de enfoques racionales para minimizar los riesgos y optimizar las prácticas destinadas a mejorar las medidas de seguridad de los pacientes^(29,44)^. Las principales conclusiones de esta revisión subrayan la importancia de comprender el comportamiento de los microorganismos relevantes en la contaminación de los textiles en diferentes regiones geográficas, teniendo en cuenta el microbioma local y las variables climáticas, como la temperatura y la humedad. Además, la revisión destaca la importancia de identificar métodos y técnicas para la manipulación y desinfección de textiles, abordando parámetros como la temperatura, el tiempo de exposición y los agentes desinfectantes más eficaces para los procesos de lavado. La revisión también abarca los recursos y materiales adecuados para el almacenamiento y el aprovisionamiento, así como la aplicación de tecnologías avanzadas en la fabricación de tejidos, con el objetivo de reducir la carga microbiana en los tejidos utilizados en el contexto sanitario.

Aunque existen revisiones similares sobre el uso de textiles como posibles fuentes de contaminación, estas revisiones se centraron únicamente en estudios que abordaban la contaminación de los tejidos, sin tener en cuenta la contaminación textil asociada a las IAAS. Otra limitación fue la ausencia de detalles metodológicos, incluida la estrategia utilizada para seleccionar los estudios potencialmente elegibles, así como los descriptores y las bases de datos empleados^(15,45)^.

## CONCLUSIÓN

La relación entre la manipulación, el procesamiento y el almacenamiento de los textiles está directamente relacionada con la presencia de contaminación por microorganismos patógenos y desempeña un papel fundamental en la aparición de IAAS en entornos hospitalarios.

La manipulación, el procesamiento y el almacenamiento inadecuados de estos materiales pueden facilitar la propagación de microorganismos, comprometiendo la seguridad tanto de los pacientes como de los profesionales sanitarios. Además, la manipulación inadecuada y la exposición continua a superficies contaminadas aumentan el riesgo de transmisión, lo que subraya la necesidad de protocolos estrictos para la gestión de los materiales textiles en entornos sanitarios, adaptados al perfil microbiológico detectado.

En este contexto, la vigilancia continua de las infecciones asociadas a la atención sanitaria es fundamental para identificar las deficiencias en los procesos de limpieza, evaluar la eficacia de los protocolos adoptados y aplicar medidas correctivas para reducir la incidencia de infecciones. Por lo tanto, es esencial implementar estrategias de control y prevención que incluyan la formación profesional continua, la mejora de los métodos de desinfección y la adopción de tecnologías más eficientes en la producción y/o descontaminación de los materiales hospitalarios, con la opción de sustituir los materiales cuando se considere necesario.

El control estricto de los equipos utilizados en el lavado y procesamiento de los textiles hospitalarios es un factor crítico para garantizar una descontaminación eficaz. Esto requiere un mantenimiento periódico, una calibración y un monitoreo microbiológico para evitar fallos operativos. Además, el almacenamiento adecuado de estos materiales es igualmente importante, ya que el contacto con superficies contaminadas después del lavado puede comprometer todo el proceso de limpieza. Por lo tanto, el almacenamiento adecuado debe cumplir con normas específicas de bioseguridad para garantizar que los tejidos estén protegidos contra la recontaminación antes de su uso.

El control de las infecciones asociadas a la atención sanitaria requiere un compromiso institucional con la vigilancia activa, la calidad de los procesos hospitalarios y el cumplimiento de protocolos estrictos, garantizando que los textiles hospitalarios se manipulen, procesen y almacenen de forma segura. En última instancia, esto reducirá los efectos negativos sobre la salud de los pacientes y mejorará la eficacia general de los servicios de salud.

## Data Availability

https://doi.org/10.48331/SCIELODATA.6DMHMC
